# Exploring staff experiences and perceptions of patient‐perpetrated violence in hospital settings: A qualitative study

**DOI:** 10.1111/jocn.17218

**Published:** 2024-05-19

**Authors:** Dana Sammut, Liz Lees‐Deutsch, Luul Ali, Jennifer Imasogie, Lavinia Nkundo, Nutmeg Hallett

**Affiliations:** ^1^ Centre for Healthcare and Communities Coventry University Coventry UK; ^2^ School of Nursing and Midwifery, College of Medical and Dental Sciences University of Birmingham Birmingham UK; ^3^ Centre for Care Excellence University Hospitals Coventry and Warwickshire NHS Trust Coventry UK

**Keywords:** exposure to violence, health personnel, patient assault, professional burnout, qualitative, verbal abuse, workplace violence

## Abstract

**Aims:**

To explore hospital staff experiences and perceptions of patient‐perpetrated violence.

**Design:**

Descriptive qualitative study.

**Methods:**

Twelve semi‐structured interviews (June–August 2022) were held with a diverse sample of hospital nurses, doctors, allied health professionals, security and a non‐clinical manager. The framework approach was used to organise and analyse data, using Attribution Theory as a theoretical lens.

**Results:**

Three themes were identified: violence as (un)predictable, violence as (un)preventable and the cumulative toll of violence. In making sense of why patients become violent, participants described different ‘types’ of aggressive patients and variably attributed behaviours to situation, disposition or a combination of both. Regardless of perceived causal factors, staff overwhelmingly appeared to view violence as predictable. Participants also reflected on the wider structural problems underpinning violence, frequently alluding to their sense of relative powerlessness to initiate change. The cumulative toll of violence was a common thread, with staff describing their acquisition of ‘resilience’ and reflecting on its role in their responses to escalating situations.

**Conclusions:**

Many hospital staff are resigned to the inevitability of violence. The concept of staff ‘resilience’ following violence is not unproblematic, having the potential to serve as a guise for acceptance and as an additional variable for which staff are held accountable. When designing strategies, organisations should ensure that accountability for violence reduction is distributed across multiple levels. This study makes a novel contribution by exploring the perspectives of multiple staff groups working across diverse hospital settings, and adds to a sparse literature on this subject in the UK.

**Implications for the Profession:**

Efforts to address violence against healthcare staff need to be power‐conscious, ensuring that accountability is distributed across multiple levels.

**Reporting Method:**

This study is reported in line with the Consolidated Criteria for Reporting Qualitative Studies (COREQ).

**Patient or Public Contribution:**

No patient or public contribution.

**What does this paper contribute to the wider global clinical community?**

- Highlighting the issue of staff feelings of powerlessness, this study draws attention to the need for organisational and senior leadership support in the context of workplace violence. Creating and implementing effective institutional support systems can be a key measure for mitigating healthcare workers' acceptance of violence as an unavoidable aspect of the job.
- The narratives presented in this study pointed to an overlap between (self‐described) resilience and internalised acceptance of violence, suggesting that the two go hand in hand. This finding underlines a need to distinguish between healthy coping mechanisms and resignation to adverse conditions.

## 1 INTRODUCTION

Workplace violence in healthcare settings is a pervasive global problem with consequences for staff, service users and organisations. While various definitions of workplace violence exist, most adopt a broad interpretation that encompasses actual and threatened physical and non‐physical abuse against staff. Physical violence (including punching, pushing, biting and using objects as weapons) typically receives greater recognition in violence reduction strategies (Rossi et al., 2023) and is similarly more likely to be reported by hospital workers (Pompeii et al., 2016). Despite this, non‐physical violence (including verbal aggression, harassment and threatening or intimidating behaviours) is far more prevalent and, according to some studies, correlates with a higher incidence of specific adverse outcomes, including staff burnout, psychological distress and intention to leave (Zhan et al., 2019). Violence perpetrated by patients and visitors is the most common type reported in healthcare settings and accounts for the majority of staff physical and psychological injuries (Occupational Safety and Health Administration [OSHA], 2015).

## 2 BACKGROUND

A systematic review and meta‐analysis by Liu et al. (2019) examined the global prevalence of violence in healthcare settings, synthesising data from 253 studies, and found that 61.9% of workers reported experiencing a form of patient‐ or visitor‐perpetrated violence in the last year. This rose to 66.2% for staff working in general hospitals and 79.4% for emergency department staff. Furthermore, recent evidence suggests that healthcare workers' exposure to workplace violence increased during the COVID‐19 pandemic (Rossi et al., 2023), which researchers attributed to reasons that continue to endure post‐pandemic (e.g. long waiting times, lack of resources and short staffing). In the United Kingdom (UK), the most recent British Social Attitudes survey found that public satisfaction with the National Health Service (NHS) is at its lowest level since the survey began in 1983, with waiting times and staff shortages accounting for most dissatisfaction (Morris et al., 2023). Similar findings were reported in the 2022 Ipsos Global Advisor survey, where 61% and 42% of respondents (global country average) respectively agreed that their local health services were overstretched and experiencing staff shortages (Ipsos, 2022).

Healthcare organisations have a duty to protect workers' physical and mental wellbeing through the provision of safe working conditions. In 2021, international interim guidance was published to address the elevated occupational hazards facing health workers during the pandemic; among a list of workplace violence‐specific recommendations, organisations were advised to assess local violence risks ‘in consultation with workers and their representatives’ (World Health Organization & International Labour Organization, 2021, p. 9). Many academic and clinical professionals advocate for stakeholder involvement (healthcare staff) in defining local needs and strategies, as evidence continues to highlight the need for workplace violence interventions that are tailored to meet the needs of different users and settings (Somani et al., 2021). Despite these recommendations, healthcare staff continue to report feeling powerless and poorly supported by the systems designed to protect them from violence (Oxtoby, 2021). Qualitative research serves an important function in capturing stakeholder perspectives, yet little has been published to explore healthcare workers' experiences in the UK.

Violence against staff is gaining traction in UK healthcare policy. Since the introduction of a national violence reduction strategy in 2021, NHS commissioners and providers are required to demonstrate their adherence to a risk‐based framework, with the goal of enhancing consistency and transparency in risk mitigation across England's health services (NHS England, 2021). Funding has also been committed to pilot the use of body‐worn cameras by ambulance staff and roll out further training for staff working in high‐risk roles, including in acute and specialist secondary care settings (NHS England, n.d.). Though many have welcomed these initiatives, some argue that more support is needed to protect workers' mental wellbeing in the aftermath of violence, and that current approaches are too reactive (Oxtoby, 2021). Also absent from these initiatives is a clear strategy for redressing the harms of deeply rooted organisational cultures that engender fear and acceptance among staff, described in a recent opinion piece as a ‘culture of hierarchy, patriarchy, and power’ that is endemic to the NHS (Fleming, 2023, p. 312).

Across three global qualitative systematic reviews of healthcare staff experiences of violence (Ashton et al., 2018; Zhang et al., 2021; Zhong & Shorey, 2022), only three of the 30 included studies were conducted in the UK, with two of the three published over 10 years ago. The significance of this literature gap lies partly in the distinctiveness of the UK's NHS compared to the healthcare models of other nations dominating the workplace violence research landscape, including the United States and Australia. It is possible that publicly funded health services engender different expectations (from both staff and the public) than private or hybrid systems, potentially shaping the dynamics of violent encounters (Pines et al., 2023). This, alongside the inevitable differences in policy and cultural norms, underpins the need for research specifically focused on the UK context. Further, much of the literature published in this area—including the three aforementioned reviews—limits it focus to single staff groups or clinical settings. Nurses, doctors, psychiatric and emergency settings are often singled out for the purposes of primary and secondary investigation (Liu et al., 2019), resulting in the omission of key perspectives across different clinical contexts. Very little published research captures the views of security staff or hospital policymakers, for example, despite their critical roles in managing violence. This study seeks to address these gaps in the extant body of knowledge.

## 3 THE STUDY

### 3.1 Aim

This descriptive qualitative study sought to address the following question: How do staff describe their experiences and understanding of patient‐perpetrated violence in hospital settings? In view of gaps in the extant literature, a qualitative approach was selected for its ability to generate in‐depth insights into the contextual nuances and diverse perspectives of staff working in various roles and settings in an NHS hospital.

## 4 METHODS

### 4.1 Design and setting

The present research is part of a larger multi‐phase, multi‐method study involving retrospective exploration of reported incidents, co‐design of a risk assessment tool for violence and subsequent feasibility testing, which initially sought to focus on violence in emergency care settings (emergency departments and/or acute medical units). Following engagement with operational processes and key stakeholders at the primary research site, we opted to broaden the study scope to capture violence across hospital settings more generally. This decision was made to reflect the pervasiveness of violence and the severity of its effects beyond emergency care, which as a setting remains disproportionately well‐represented in violence research.

Semi‐structured interviews were held with a convenience sample of hospital staff at a large NHS teaching hospital in England (containing 1005 beds and employing over 8500 staff). At the time of data collection, amidst ongoing challenges in the pandemic aftermath, managers faced difficulty releasing clinical staff for interviews due to workforce shortages and increasing patient admissions. Despite this, the study drew the interest of staff working in various capacities throughout the hospital.

### 4.2 Recruitment

Clinical leads at the hospital were purposively approached by email according to their role, to notify them of the research and invite them to cascade recruitment materials to prospective participants (hospital staff) via their departmental mailing lists. Details about the study were also shared during meetings with an established ‘violence and aggression’ operational group at the hospital, comprising senior hospital staff with a role or interest in violence prevention. Finally, information about the study was spread via word of mouth. All staff (including non‐patient‐facing) who self‐identified as either witnessing or directly experiencing patient violence were eligible to participate.

Staff expressed their interest by approaching a member of the research team (LLD) via email, listed on the recruitment materials, or face‐to‐face after details of the study were shared during operational group meetings and/or spread via word of mouth. All expressions of interest were then followed up by DS via email, which contained a brief introduction and copies of the participant information sheet (PIS) and consent form. Prospective participants were asked to respond to the email to confirm their continued interest and indicate their availability; subsequent communication continued via email in order to coordinate interview arrangements.

The PIS explained that participants could opt for an in‐person interview, held in private rooms across the hospital, or a virtual interview via Microsoft Teams© or Zoom©. It also advised that staff attending virtually should join from a private location (within the hospital or elsewhere). However, this was left to the discretion of the participants, and in the case of disturbance or interruption, the interviewer would offer to pause or stop the interview. Staff who opted to interview virtually (*n* = 5) were asked to sign and return a copy of their consent form to DS via email; staff attending in‐person interviews (*n* = 7) were given the option of returning written consent in either digital or physical format.

Of the 16 staff who expressed interest in the study, 12 went on to participate; no reasons were given by those who opted not to continue after their initial expression of interest. Interviews were held between June and August 2022.

### 4.3 Data collection

All interviews were conducted by the lead author (DS, female), who is a registered nurse and research associate, externally contracted, with experience in qualitative interviewing. The interviewer had no prior relationship with any of the study participants. At the start of each interview, the interviewer introduced herself by name, role and background, and provided a definition of violence for the purposes of the study, adapted from Hallett (2018): ‘Any non‐verbal, verbal, or physical behaviour exhibited by a person which makes it difficult to deliver good care safely. Verbal includes the use of inappropriate words; non‐verbal includes behaviour that causes distress and/or constitutes harassment; and physical includes the intentional application of force against another person without lawful justification, resulting in physical injury or discomfort’.

The interview questions were informed by literature reviews conducted in an earlier phase of the wider study (Sammut et al., 2022; unpublished) and clinical practice expertise. Three research team members (DS, LLD, NH) collaborated to design the interview guides, with separate versions developed for patient‐facing staff (Data S2) and service leads (Data S3), to ensure we were inclusive of the potential to recruit from a diverse participant population. For participants whose role aligned with both descriptors, a hybrid approach was taken where questions from both guides were used. The amount and focus of information offered by participants influenced whether all questions were asked, and the order in which each interview proceeded. At the end of each interview, participants were asked to share their thoughts about violence risk screening, with these data analysed separately to inform a separate arm of the larger study.

The interviewer made memos during and after each interview, respectively capturing points of interest and observed data for further discussion and reference. One hour was scheduled for the interviews, which were audio recorded and then transcribed by an external provider.

### 4.4 Data analysis

Data were analysed using the framework approach (Ritchie & Spencer, 1994) involving five steps: (1) data familiarisation, (2) framework construction, (3) indexing and sorting, (4) data display and summary and (5) mapping and interpretation. This method was chosen for its pragmatic and systematic way of organising large qualitative datasets, facilitating collaboration among multiple analysts.

Four team members were involved in steps 1–5, including the lead researcher (DS) and three master's students (LA, JI, LN). Following familiarisation with all 12 transcripts, LA, JI and LN each took four transcripts to begin identifying preliminary codes. This was an inductive process, producing descriptive codes that captured surface‐level meaning. Once complete, the three members met with DS to discuss and reflect on their early outputs, before collaboratively grouping the preliminary codes to produce initial framework categories and subcategories (Data S4). The framework was transferred to a digital spreadsheet and piloted using data from across all 12 transcripts; after further discussions, it was agreed that no revisions were required, and indexing and sorting continued until the full dataset was captured within the framework. LA, JI and LN each completed indexing and sorting for their four allocated transcripts; DS then reviewed all indexed data against the 12 transcripts.

**FIGURE 1:**
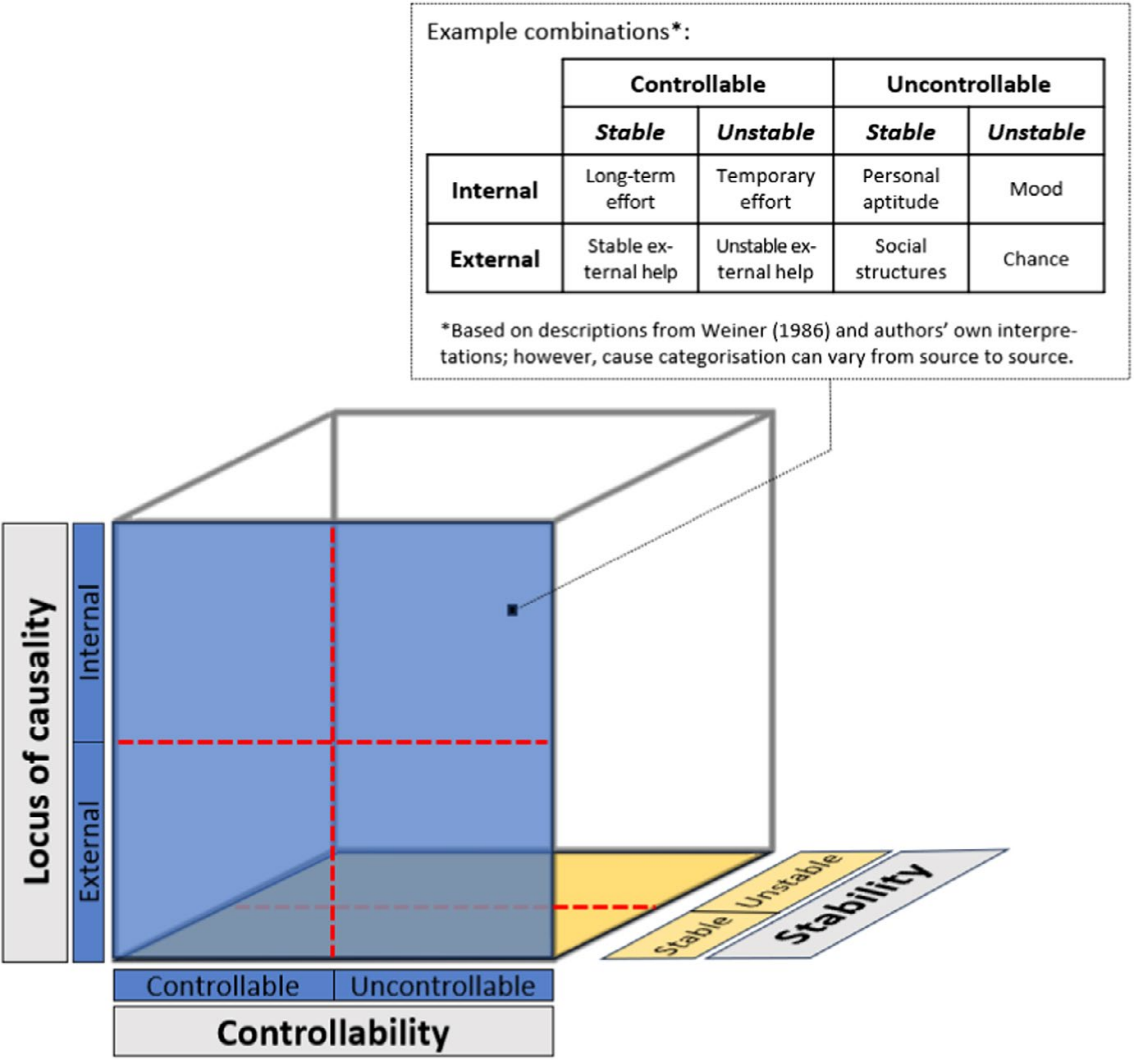
Attribution Theory dimensions and example combinations. [Colour figure can be viewed at wileyonlinelibrary.com]

Next, we revisited the framework to look for patterns of shared meaning across each group of initial themes and categories. To facilitate this, summaries of the indexed data were produced using a matrix, with participants organised into rows and the framework categories and subcategories organised into columns. The resulting summaries were reviewed and reduced, through the process of abstraction, to produce refined categories. We did not set out to use any specific theory ahead of data collection and analysis; instead, through the process of engaging with the data, we identified the relevance of Attribution Theory (Weiner, 1986) in relation to some of our refined categories. According to this theory, individuals try to make sense of the world around them by attributing causality to the actions and events they observe. There are three primary dimensions of attribution: *controllability*, *stability* and *locus*. Respectively, these relate to the degree of control individuals have over outcomes, the degree to which causes fluctuate over time, and whether causes are internal/dispositional (relating to internal characteristics or personal qualities such as personality) or external/situational (relating to external circumstances such as work environment) (Figure 1). At this stage, we decided to revisit the full dataset and review our summary matrix using Attribution Theory as a theoretical lens. New codes and analytical memos were also created.

Within the final step, we reviewed the relationships among all components of the analytical framework, to define (and interrogate) the conceptual coherence across each iteration. To define core concepts, we reviewed the data in a more theory‐ and context‐aware analytic mode, reflecting upon the insights offered by Attribution Theory as well as our own assumptions and biases as researchers with clinical experience. This stage involved revisiting the whole dataset, together with our collective memos and notes, and mapping our analytical interpretations against each transcript. At the end of this process, each framework component was assigned a final theme name to reflect its distinct central organising concept.

### 4.5 Ethical considerations

Approvals for this study were granted by the University Hospitals Coventry and Warwickshire NHS Trust Research and Development Department (reference: GF0480).

### 4.6 Rigour

The processes outlined above demonstrate rigour through adherence to Lincoln and Guba's (1985) quality criteria. The involvement of multiple researchers in developing and applying the analytical framework, together with detailed and transparent documentation of the steps involved, ensured credibility and dependability. Drawing on theory during analysis enhanced the transferability of findings to other contexts, while the analysts' efforts to critically reflect—individually, in the form of reflexive note‐writing, and collectively by way of regular dialogue—enhanced confirmability. This study is reported in line with the Consolidated Criteria for Reporting Qualitative Studies (COREQ) (Tong et al., 2007) (Data S1).

## 5 FINDINGS

### 5.1 Participants

Participants (*n* = 12) were predominantly registered health professionals (*n* = 10): physiotherapists (*n =* 4), nurses (*n* = 3), doctors (*n* = 2) and a dietitian (*n* = 1). Of these ten, five worked in senior roles involving both patient‐facing and team/service lead work, and one worked in a non‐patient‐facing senior position. The remaining two participants were a member of security staff (patient‐facing role) and a non‐clinical manager (non‐patient‐facing role). All participants identified themselves as having a role that involved managing and/or witnessing patient violence. The interviews ranged from 35 min to 1 h and 10 min in length (mean length: 51 min).

### 5.2 Thematic findings

The analysis yielded three themes: violence as (un)predictable, violence as (un)preventable and the cumulative toll of violence.

#### 5.2.1 Violence as (un)predictable

In making sense of why patients become violent, participants often described different ‘types’ of aggressive patients and variably attributed patients' behaviours to situation, disposition or a combination of both. This approach reflects the basic tenets of Attribution Theory, which holds that when trying to make sense of others' actions, individuals attribute the behaviour to internal dispositions or external situations. Participants described a range of patient‐related risk factors including drug and alcohol intoxication, cognitive impairments, pain and fear. These factors were generally framed as something incidental, transient or beyond the patients' control, suggesting an external locus of causality. For example, experiences in certain specialties highlighted aggressive encounters that were reflective of patients' current health condition(s), such as dementia or injury‐related cognitive changes, which staff considered to indicate a lack of intent. Participants similarly reflected on the way patients and their relatives can feel out of control, whether due to a physical condition or wider personal or social circumstances, leading them to lash out in an attempt to regain some sense of autonomy. This interpretation of behavioural escalation as a desperate bid for control, rather than unprovoked hostility, was often accompanied by expressions of empathy from participants:

Relatives, if the phones aren't answered, or they're not happy with the discharge plan… But for relatives, you can tell [those] who have been struggling at home, trying to cope for a member of family, and they're just trying to vent or make it clear that it hasn't been an easy dynamic. Then you can see why they get frustrated.

P2 (Physiotherapist & Team/Service Leader)

While situational factors were frequently discussed, dispositional attributions were also considered, albeit to a lesser extent. When describing inherent personality traits as a cause of aggression, participants often qualified their statements by relating these traits to broader external circumstances. Here, staff alluded to the myriad of psychosocial factors at play: for example, the ways in which individuals and communities are impacted by systemic pressures stemming from gang violence, social stigmas or personal histories of abuse. These qualifications suggested a nuanced interplay between dispositional and situational factors, and provided further evidence of participants' proclivity to empathise with aggressive patients, whether as a means of making sense of violence or enabling a more considered response:

A lot of our patients, they've already been spoken to, perhaps not most sensitively, by other healthcare professionals. So whether that's the [general practitioner] who referred them, or it could be a trauma and orthopaedic specialist. But a lot of patients say that the way they're spoken to about their weight is not helpful. […] They already come with that kind of dread and fear, and they fear that we're going to judge them so much. Sometimes, [female patients] come into the room and they're very on the defence. And then, when you start showing a bit of empathy and a bit of compassion, you're listening to them, and they completely flip and open up.

P4 (Dietitian)

In some cases, deep‐seated personality traits or characteristics, such as entrenched chauvinistic attitudes, were mentioned in the context of behaviours that participants considered to be unalterable or beyond the staff's ability to reason with. These behaviours were met with pragmatic solutions, such as accommodating patients' gender preferences (both anticipated and realised), owing to limited alternatives when faced with such sources of hostility. When alluding to dispositional attributions, participants often spoke with a sense of resignation and powerlessness. At the same time, these attributions seemed to present a means for staff to distance themselves from the situations they were describing, as though to reconcile their efforts to accommodate patients' needs with the continuous aggression they received in response. For example, after describing staff efforts to address the behaviour of a patient who was physically violent ‘every day […] over a period of months’, P8 (Physiotherapist) summarised by saying, ‘Did it change anything? No. He continued doing it. That was his personality’.

Yet, regardless of perceived causal factors, staff overwhelmingly appeared to view violence as predictable. This view was sometimes communicated explicitly, with participants talking about behaviour escalation as something formulaic with a predictable trajectory. For example, staff noted how common sources of patient frustration—such as being denied the opportunity to smoke a cigarette—would often escalate into more pronounced aggression, with patients exhibiting a visible build‐up that eventually culminates in an explosive outburst. In other instances, despite using language such as ‘unpredictable’ and ‘without warning’, participants recalled incidents that could be described as having clear warning signs, or which featured characteristics that the participant had elsewhere recognised as being violence risk factors. One notable account involved a patient exhibiting intimidating behaviour during a face‐to‐face clinic appointment, with the participant (P4, Dietitian) recalling that they were caught off guard due to there being no warning signs documented in the patient's notes. Later in the interview, the participant recalled that this patient had a known history of cannabis‐induced psychosis and paranoia, and recognised that, in hindsight, this information could have raised a cause for concern.

A number of explanations were offered for why staff miss the warning signs of escalating behaviour, often focusing on the fast pace and competing demands of clinical work, as well as individuals' skill and experience. Nevertheless, these accounts generally communicated a belief that staff *would* be able to predict violence with the right conditions and tools:

As an older person, I've been through quite a lot of conflict with my wife and children and whatever. And you read the signals and you read it in other people. So, if I saw you getting frustrated, I probably would deal with you a certain way, whereas our younger members of staff may not yet have that skillset. […] You must be able to see the build‐up to a violent exchange in any given scenario. And what I'd really like to give our staff is, ‘these are the tension indicators to be looking for, and this is potentially what you can do to defuse that situation’.

P11 (Non‐clinical Manager)

These excerpts illustrate the breadth of participants' experiences and interpretations of patient violence as a complex and multifaceted issue. Though most seemed to regard violence as predictable to some extent, perspectives on its preventability varied considerably.

#### 5.2.2 Violence as (un)preventable

Considering the precursors and/or triggers for behavioural escalation, participants often reflected on the impact of wider systems and structures which they perceived to precipitate violence. These included departmental‐ and organisational‐level issues, such as lack of staff and long waiting times, as well as broader socio‐political factors. Some participants described how increasing patient acuity, together with the aftermath of COVID‐19 and rising mental health needs, had stressed the healthcare system beyond its capacity, leaving hospitals ill‐equipped to meet changing demands. Operational inefficiencies were also highlighted, such as appointments given in error, or services becoming unavailable at short notice, leading patients to enter the healthcare setting prepared for confrontation. This perceived strain on the NHS was identified by some staff as contributing to a decline in patient tolerance:

Certainly, in terms of the number of complaints and dissatisfaction, that seems to have gone up massively after COVID. Initially there was obviously the clapping for the NHS and people were so happy with the care that was provided, and ‘you angels’ and this kind of thing. And then obviously since that, when they realised that it's been another two years and they've still not got their operation or whatever, that makes people really dissatisfied with the service that we are providing.

P6 (Nurse & Team/Service Leader)

Throughout these reflections, participants alluded to hierarchical structures, both within and beyond their professional roles, and often positioned themselves as actors for change in the context of these hierarchies. For some, conversations about preventing violence were overshadowed by an *us‐*versus*‐them* rhetoric, communicating a sense of subordination and a perceived inability to initiate change. The frustration of these staff towards senior managers and decision‐makers within the hospital was marked by a perception that decisions (or lack thereof) seemed to undermine those on the ground in their attempts to manage or prevent violence effectively:

There needs to be something else, but none of us are mental health trained, and they put us frontline. And that's a big thing that we've been trying to push for, but that means more money.

P12 (Security)

When speaking from the ‘bottom‐up’ perspective, participants often indicated a sense of reluctant acceptance, as though resigned to the inevitability of violence. P12 (Security) concluded: ‘Our job is literally to get attacked, that's how it seems at the moment’. In other instances, despite taking a more optimistic stance on violence prevention, participants' views on current and hypothetical strategies were placed in the context of wider limiting variables beyond their control. Many of these discussions centred around a scarcity of resources, such as specialised cohort areas for vulnerable patients, while the issue of understaffing predominated many participants' reflections on the feasibility of preventing violence.

Beyond the systemic barriers to reducing risk, participants considered the issue of accountability and its distribution across multiple levels. Often, these reflections seemed to reiterate the view that staff members' capacity to prevent violence is limited by their position within the organisational hierarchy, with each eventually reaching a ceiling:

We were able to escalate it to our managers, who then escalated it to the ward manager. Unfortunately, the ward manager did everything they could, but there's only so much a ward manager can do. They then escalated it to the consultant. Unfortunately, the consultant was not there every day, and so they didn't see the violence that happened. And so, they were not as supportive at the time.

P8 (Physiotherapist)

Similar views were expressed by participants speaking from the ‘top‐down’ perspective, with staff in positions of authority holding themselves accountable for the safety of those under their purview. One doctor (P9), emphasising the need to support junior staff, recalled stepping in to protect junior colleagues who were less confident and more affected by patient aggression, indicating an assumed duty of care. Similarly, P11 (Non‐clinical Manager) talked of managerial culpability in providing frontline staff with adequate tools to prevent violence, highlighting the cascading effects of decisions made by those at the ‘top’ of the hierarchical divide.

Others suggested a reciprocal interdependence between patient‐facing staff and senior decision‐makers, with each having the power to limit or bolster the other's efforts to prevent violence. One physiotherapist and team/service leader (P3) reflected on the impacts of staff underreporting, noting that it complicates the ability of those in leadership positions to identify patterns and implement appropriate strategies accordingly. Conversely, participants whose roles were predominantly or entirely patient‐facing often voiced frustration over senior managers' apparent lack of awareness of the extent of violence. These communication gaps between different levels of the organisational hierarchy seemed to shape staff responses and perceptions of violence as something that can be mitigated:

I think some of the problem is that people don't report because they don't see anything happen at the end of that […]. So, it's almost like, well, what's the point in reporting it, because they don't do anything anyway? I mean clearly things are happening behind the scenes, but the staff don't always know that, so there's a feeling that there's a lack of support, so what's the point. Whereas actually I know the trust is looking at how they can improve that. For me, I'm always saying the more we report something, they're going to have to do something about it, because if we don't report it, it's not a problem. If all of a sudden our reporting goes through the roof it's like, hang on, we've got a spike here, there's something going on. We need to look at this.

P1 (Nurse & Team/Service Leader)

Despite a common theme of powerlessness, patient violence generally seemed to be regarded by participants as preventable *in theory*. Though many acknowledged violence to be a collective problem, staff were acutely aware of their limited capacity as individuals to effect change. Similarly, while dispositional factors were alluded to occasionally, narratives surrounding violence preventability were overwhelmingly focused on external causes (often beyond participants' control).

#### 5.2.3 The cumulative toll of violence

The immediate impacts and wider consequences of patient violence were discussed at length by interviewees. Many recognised that staff experience violence in different ways, influenced by factors such as the clinical setting, staff group, ethnicity, age and gender, affecting not only the frequency of violence but also the severity of its effects.

While non‐physical violence was touched upon throughout the interviews, it was clear that many staff viewed it as less significant than physical assault. Expressing a sentiment echoed by others, one doctor (P10) stated, ‘If someone was verbally abusive to me, I probably wouldn't even think about [reporting it]’, indicating a threshold for what constitutes report‐worthy violence. When physical assault occurred, its perceived severity also factored into the way staff considered and responded to it. In one excerpt, P7 (Physiotherapist) recounted an incident involving sexual assault which resulted in significant emotional distress but no physical injury. On reporting this to the ward's nursing staff, the participant was met with a dismissive response, which they later attributed to the frequency and consequent normalisation of violence within that setting. Supporting this idea, another participant stated:

If you had to fill out a [report] every time a patient grabbed you inappropriately, you wouldn't get anything else done, so to speak […]. But it doesn't make it right, but obviously the significant injuries do get reported. But I think a lot of staff, experienced staff, do take it that it is part and parcel of their day‐to‐day work.

P5 (Nurse & Team/Service Leader)

These accounts point towards a broader dismissiveness of violence that is not archetypal, with certain types of aggression, often from patients lacking capacity, perceived as benign or inconsequential. In another example, after initially giving little weight to their experiences involving ‘elderly patients with dementia [who] give you a rap on the knuckles’, P3 (Physiotherapist & Team/Service Leader) later qualified: ‘I imagine when you're having that ongoing tirade of verbal or physical smaller incidents, but repeatedly over a long period of time, that will have a huge impact’. This addendum followed an acknowledgement by P3 of their outsider perspective, holding a predominantly non‐patient‐facing role. In the context of participants' frequent references to non‐reporting—including P3's account of its impacts on their role—it is not surprising that these forms of violence become obscured by a more dominant narrative.

Reflecting on staff responses to violence and aggression, some participants alluded to dispositional factors, suggesting that professionals' ability to manage behavioural escalation is determined by their (largely unalterable) personal characteristics. Among these were personality, long‐held attitudes and self‐confidence, which were often presented as innate and unteachable. While some participants described these qualities in a neutral way—for example, acknowledging that personality clashes are unavoidable—others considered how these factors might shape a uniformity in staff responses to aggression, albeit not always producing the same result. P9 (Doctor) reflected on their inclination towards assertiveness, recognising the potential of this approach to escalate situations further, but framed it as a strategic decision reflective of personal agency. Other participants similarly associated self‐confidence with an ability to exert greater control in challenging situations, with many seeing it as something valuable that is acquired over time:

As a young female, as soon as I entered the job, I experienced inappropriate sexual comments, and I didn't know what to say or do or how to react. Now I know to shut it down, report it if needed, and immediately say to the patient, there and then, ‘that's not appropriate’. But it's having the experience and confidence to say that and think, actually, I can say that to a patient, and actually, this is not acceptable.

P8 (Physiotherapist)

Many staff reflected on how their responses had changed for the better following exposure to violence, often attributing this to improved resilience. However, the concept of resilience seemed to present a paradox, with some narratives suggesting that resilience (equated to being less emotionally affected by incidents) is both strengthened and weakened by exposure to abuse. The emotional toll of violence was a common theme across the interviews, with many suggesting that staff who have been worn down by continuous exposure respond less positively to escalating situations. As a causative factor, this emotional fatigue was often framed as something situational, though nevertheless beyond staff's immediate control. One participant (P4, Dietitian) recalled witnessing consultants ‘losing their temper’ with patients, positing that ‘they were just so sick of abuse, day in, day out’. Others reflected on the changes they witnessed in themselves and others due to this exposure, including heightened guardedness, which ultimately impeded their ability to effectively manage behavioural escalation.

Meanwhile, it was also often held that experienced staff are less affected by their encounters with aggression, with some seeming to draw parallels between resilience and the ability or willingness to tolerate violence. On one hand, resilience was viewed as a strength, a by‐product of professional experience that rendered staff less emotionally vulnerable to incidents of aggression. This perception underscores an implicit expectation within the healthcare sector that dealing with violence is part of the job, a sentiment echoed by many interviewees. However, it was also implied that staff who are more used to (and less bothered by) violence are more likely to accept it as the norm. For example, P6 (Nurse & Team/Service Leader) suggested that for staff facing frequent violence, it becomes a nonissue, likened to ‘water off a duck's back’. This form of adaptation, where the line between resilience and resignation becomes blurred, is further illustrated by the following excerpt:

One the juniors was on receiving end of a horrible thing. They felt very aggrieved by it, very upset by it. As if [to say], why aren't they supporting us? Why are we on the receiving end of that behaviour? We shouldn't have to treat him. [But] we do have to still treat him as best we can. We've got to work with these patients. So, I think there's a certain zero tolerance of some of the younger members of staff for it. And whether that… I don't know, it's a difficult dynamic.

P2 (Physiotherapist & Team/Service Leader)

These narratives point to an overlap between (self‐described) resilience and internalised acceptance of violence. Despite participants presenting varied interpretations of their own and their colleagues' responses to aggression, they collectively depicted a cumulative effect that can manifest dispositionally (personality or attitude changes over time) or situationally (intermittent behavioural changes owing to burnout).

## 6 DISCUSSION

This study aimed to explore hospital workers' experiences and understanding of patient‐perpetrated violence. Our analysis identified three themes: *violence as* (*un*)*predictable*, *violence as* (*un*)*preventable*, and *the cumulative toll of violence*. In many ways, these findings reflect the narratives presented in extant international literature on workplace violence.

Regarding violence predictability, participants in our study alluded to various internal (dispositional) and external (situational) causes of violence. These attributions, which were predominantly external, seemed to provide a focal point around which staff made sense of their experiences and responses. In a qualitative meta‐synthesis, Ashton et al. (2018) indicated that emergency department staff often ascribed patient violence to aggressor characteristics such as capacity or perceived intent, which subsequently influenced the staff's emotional and behavioural responses. The relationship between causal attributions and staff‐patient interactions was also explored by Enosh et al. (2021), who postulated that conflict situations are more likely to escalate when clinicians attribute causality to aggressor disposition, owing to issues of blame and stigma. In a qualitative study, Pines et al. (2023) reported that external attributions enabled healthcare staff to feel and demonstrate a greater level of empathy, sometimes leading to allowances for challenging patient behaviours. Interactional dynamics are difficult to quantify, and further research is needed to explore the relationship between these variables. However, while it may be beyond the scope of most studies to establish causality, the value of applying social‐psychological literature to workplace violence research lies beyond this endeavour.

Theory can play an important role in lessening the reductionism of complex social phenomena, informing strategies that consider the reciprocal influences between individual behaviours and broader social environments, rather than attempting to target disparate behaviours with an assumption of uniformity. See Pines et al. (2021) for an example of a theoretically‐informed workplace violence intervention. On the issue of workplace violence, reductionism can be seen in strategies that treat the violent interaction as something transient or episodic, with little regard for the wider context that shapes its development and after‐effects. This is not to say that clinically focused interventions (such as staff de‐escalation training) have no value, but instead that, as a standalone strategy, their focus is often too narrow to produce meaningful long‐term effects. This idea is supported by a recent systematic review of workplace violence interventions, which found that standalone staff education and training strategies were ineffective in reducing violence against nurses (Somani et al., 2021). The authors suggested that the main shortcoming of staff‐focused strategies is their failure to address the behaviour of aggressors. While not unreasonable, this explanation rests on the same assumption that violence is incidental, and fails to account for conditions outside of the staff‐patient interaction (e.g. organisational structures and cultures). Within the confines of an interaction, the predictability of violence might be a simple variable to isolate, but this only addresses part of the problem.

We explore the broader structural components of workplace violence in our second theme, which moves from predictability to preventability. Staff narratives communicated a sense of powerlessness that was precipitated by inadequate organisational support and rigid professional hierarchies, leaving many resigned to ‘inevitable’ violence. Similar findings are reported elsewhere (Ashton et al., 2018; Wolf et al., 2014). In their qualitative study, Wolf et al. described American emergency nurses as having ‘an expectation of violence’ (p. 307) that was reinforced by cultures of acceptance, administrative bureaucracy and societal complacency towards violence affecting nurses. Further, Drach‐Zahavy et al. (2012) found an association between staff feelings of powerlessness and other negative self‐perceptions, including a low sense of professional adequacy and efficacy, which may in turn affect staff performance, coping and willingness to engage in violence reduction efforts (Chang et al., 2022). Similar correlations are seen between staff emotional fatigue (or burnout) and susceptibility to violence, with each potentially contributing to the other (Pina et al., 2022). These invisible snowball effects are often poorly recognised—or at best, compartmentalised—in workplace violence policies and strategies, resulting in solutions that rely disproportionately on the efforts of frontline workers. This fact is reflected in the shame and self‐blame responses of staff who experience violence, and to some extent, also manifests in the resilience rhetoric that we discuss in our third theme.

The term ‘cumulative toll’ is used in the final theme to represent the short‐ and long‐term effects of violence on staff, with repeated exposure often associated with progressive (and sometimes dissonant) changes in temperament. Participant narratives seemed to suggest that experienced staff are simultaneously more resilient and more internally accepting of violence. In other words: *ability* and *willingness* to tolerate violence go hand in hand. Similar patterns were observed by Freedman et al. (2018), who argued that ‘hegemonic norms and beliefs’ (p. 116) reinforce the normalisation of violence in health settings, causing subtle shifts in staff attitudes (and eventually behaviours) over time. This evolution in the way professionals exercise their ‘micropractices of power’ has been associated with the growing gap between formal policy expectations and the realities of clinicians' daily practice (Gilson et al., 2014, p. iii51). Moreover, perceptions of organisational inaction or indifference to patient violence may reinforce staff beliefs that it should be tolerated (Ashton et al., 2018). The concept of staff ‘tolerance’ has been discussed at length in the literature, though interpretations of what this entails seem to vary, with some using the term to denote unhealthy acceptance (Richardson et al., 2018) and others to describe staff passiveness or acquiescence in response to aggression (Dadashzadeh et al., 2019). This dual meaning was also reflected in our study's findings, with the latter interpretation usually implied by participants to represent a lack of confidence that diminishes with experience, while the former, conversely, increases. A third interpretation might also be used to explain the apparent relationship between resilience and internalised acceptance, whereby staff develop a healthy disregard for violence, either consciously or subconsciously, in order to be able to ‘get on with the job’.

The question of how much tolerance (if any) may be healthy for staff is unclear, and evidence on the subject is conflicting. Staff non‐reaction or ‘tolerance’ has been recognised as an adaptive and protective coping strategy in some contexts (Fan et al., 2022), although other studies have associated it with reduced job satisfaction (Dadashzadeh et al., 2019). Similarly, while violence trivialisation was associated with an overall increase in staff psychological consequences in a cross‐sectional study by Geoffrion et al. (2017), the inverse was true for male healthcare workers. Various mechanisms have been proposed to explain these associations, with confidence (Hanson et al., 2015) and mental resilience (Kim et al., 2022) often examined as mediators. However, many of these studies analyse the correlations between a limited selection of variables, and as such, are unable to account for other personal resources and skills (such as empathy and communication competency) or wider systems factors (such as organisational responsiveness) that have been described elsewhere as protective strategies (Yao et al., 2021). Further exploration of these dynamics could help to explain these disparate findings, and shed light on the interactions between cumulative stressors and staff coping mechanisms.

Resilience has been conceptualised in a number of ways (Luthar et al., 2000), and it is used here broadly to denote positive adjustment following adversity. However, as illustrated in our final theme, the term often carries connotations that assume an alignment with personal attributes and values. The idea of resilience as a panacea—endorsed by many healthcare workers—rests on the assumption that its acquisition follows a linear and uniform process, with setbacks attributable primarily to underexposure and/or inherent personality traits (Luthar et al., 2000). This framing of resilience is both inaccurate and antithetical to the goal of creating supportive and adaptable work environments. Studies on resilience in healthcare workers describe a complex interplay between psychological factors—including personal and professional identity (Winkel et al., 2018) and the sequelae of personal traumas (Donovan et al., 2021)—social support, and organisational factors (Van Heugten, 2013). That violence affects staff resilience in different ways is therefore unsurprising. Further, the expectation that staff should ‘toughen up’ can feed into acceptance culture, heighten shame responses, and ultimately deflect attention from the systemic factors that precipitate violence. While personal resilience is undoubtedly important in this context, narratives that overemphasise its role (or underestimate its complexity) risk amplifying the conditions that hinder its acquisition.

### 6.1 Strengths and limitations

Our findings draw from the narratives of a self‐selected sample of participants recruited from a single hospital. To protect participants' anonymity, we omitted certain demographic information (including gender, current clinical area and specific job/role descriptors) when describing the sample, limiting interpretations about the significance of these characteristics. However, the diversity of our sample—representing four clinical professions and two separate non‐clinical roles—enhanced data richness by providing greater variation across the experiences explored. This was a particular strength of the study, as much of the evidence in this area focuses exclusively on individual professional groups (largely medical and nursing), with very little being published on the experiences of allied health professionals and security staff. While a larger sample size would have been helpful for conducting an in‐depth cross‐case analysis of participant perspectives, this was not the goal of this study, and the narratives we obtained provided sufficient information power to identify patterns and scrutinise meaning (Malterud et al., 2016).

## 7 CONCLUSION

This study explored the intricate landscape of workplace violence within hospital settings, revealing three pivotal themes relating to the predictability and preventability, as well as the cumulative toll, of violence. The findings align with international literature, emphasising the significance of external attributions of violence and cautioning against over‐reliance on staff‐focused interventions. The study underscores the need for comprehensive approaches that address not only individual coping strategies but also the broader organisational cultures and support systems. As healthcare policy endeavours to mitigate workplace violence with reduction strategies and staff training, it becomes increasingly evident that safeguarding healthcare workers' mental well‐being and fostering safer work environments both require a holistic perspective. To protect staff, future research and policy initiatives must prioritise understanding and dismantling the deep‐rooted cultural factors that perpetuate fear and acceptance of violence among healthcare staff, ensuring that the strategies in place truly prioritise the safety and resilience of those on the frontlines. When designing strategies, organisations should ensure that accountability for violence reduction is distributed across multiple levels. While the UK has taken notable strides in developing strategies to curtail violence against healthcare staff, it is important that future research and evaluation commit to addressing the priorities of the very staff these strategies aim to protect.

## AUTHOR CONTRIBIUTIONS

**Dana Sammut**: Conceptualisation, methodology, formal analysis, investigation, data curation, writing—original draft, writing—review and editing, visualisation, supervision, project administration. **Liz Lees‐Deutsch**: Conceptualisation, methodology, formal analysis, writing—review and editing, supervision, project administration, funding acquisition. **Luul Ali**: Formal analysis, writing—review and editing. **Jennifer Imasogie**: Formal analysis, writing—review and editing. **Lavinia Nkundo**: Formal analysis, writing—review and editing. **Nutmeg Hallett**: Conceptualisation, methodology, data curation, writing—review and editing, supervision, project administration, funding acquisition.

## CONFLICT OF INTEREST STATEMENT

A member of the research team is a Board Trustee of the Clive Richards Foundation. The Principal Investigator to whom the funding was awarded is not a trustee or related to a trustee. Charity funding is agreed in a two‐stage process: (1) finance committee and (2) the board of seven trustees. Trustees with a potential conflict of interest must abstain from participating in the decision‐making process in awarding funding. Finances were paid to the University of Birmingham for the Principal Investigator and Research Associate working on this study. No payments (salary or other) were paid to the trustee.

## Supporting information


Data S1.

Data S2.

Data S3.

Data S4.

## Data Availability

The data supporting this study cannot be made available for ethical reasons.

## References

[jocn17218-bib-0001] Ashton, R. A. , Morris, L. , & Smith, I. (2018). A qualitative meta‐synthesis of emergency department staff experiences of violence and aggression. International Emergency Nursing, 39, 13–19. 10.1016/j.ienj.2017.12.004 29326038

[jocn17218-bib-0002] Chang, Y.‐C. , Hsu, M.‐C. , & Ouyang, W.‐C. (2022). Effects of integrated workplace violence management intervention on occupational coping self‐efficacy, goal commitment, attitudes, and confidence in emergency department nurses: A cluster‐randomized controlled trial. International Journal of Environmental Research and Public Health, 19(5), 2835. 10.3390/ijerph19052835 35270527 PMC8910583

[jocn17218-bib-0003] Dadashzadeh, A. , Rahmani, A. , Hassankhani, H. , Boyle, M. , Mohammadi, E. , & Campbell, S. (2019). Iranian pre‐hospital emergency care nurses' strategies to manage workplace violence: A descriptive qualitative study. Journal of Nursing Management, 27(6), 1190–1199. 10.1111/jonm.12791 31104356

[jocn17218-bib-0004] Donovan, E. , Santer, M. , Morgan, S. , Daker‐White, G. , & Willcox, M. (2021). Domestic abuse among female doctors: Thematic analysis of qualitative interviews in the UK. British Journal of General Practice, 71(704), e193–e200. 10.3399/BJGP.2020.0795 PMC7909912 33558329

[jocn17218-bib-0005] Drach‐Zahavy, A. , Goldblatt, H. , Granot, M. , Hirschmann, S. , & Kostintski, H. (2012). Control: Patients' aggression in psychiatric settings. Qualitative Health Research, 22(1), 43–53. 10.1177/1049732311414730 21743032

[jocn17218-bib-0006] Enosh, G. , Freund, A. , Goldblatt, H. , Drach‐Zahavy, A. , Guindy, M. , & Ofer‐Bialer, G. (2021). Whose fault is it? Attribution of causes of patient violence among exposed and unexposed community‐based family physicians. Health & Social Care in the Community, 29(1), 175–184. 10.1111/hsc.13080 32627279

[jocn17218-bib-0007] Fan, S. , An, W. , Zeng, L. , Liu, J. , Tang, S. , Chen, J. , & Huang, H. (2022). Rethinking “zero tolerance”: A moderated mediation model of mental resilience and coping strategies in workplace violence and nurses' mental health. Journal of Nursing Scholarship, 54(4), 501–512. 10.1111/jnu.12753 34866319

[jocn17218-bib-0008] Fleming, S. (2023). With sexual harassment or assault, what you permit, you promote. BMJ, 381, p1117. 10.1136/bmj.p1117 37220937

[jocn17218-bib-0009] Freedman, L. P. , Kujawski, S. A. , Mbuyita, S. , Kuwawenaruwa, A. , Kruk, M. E. , Ramsey, K. , & Mbaruku, G. (2018). Eye of the beholder? Observation versus self‐report in the measurement of disrespect and abuse during facility‐based childbirth. Reproductive Health Matters, 26(53), 107–122. 10.1080/09688080.2018.1502024 30199353

[jocn17218-bib-0010] Geoffrion, S. , Goncalves, J. , Boyer, R. , Marchand, A. , & Guay, S. (2017). The effects of trivialization of workplace violence on its victims: Profession and sex differences in a cross‐sectional study among healthcare and law enforcement workers. Annals of Work Exposures and Health, 61(3), 369–382. 10.1093/annweh/wxx003 28355455 PMC6824521

[jocn17218-bib-0011] Gilson, L. , Schneider, H. , & Orgill, M. (2014). Practice and power: A review and interpretive synthesis focused on the exercise of discretionary power in policy implementation by front‐line providers and managers. Health Policy and Planning, 29(Suppl 3), iii51–iii69. 10.1093/heapol/czu098 25435536

[jocn17218-bib-0012] Hallett, N. (2018). Preventing and managing challenging behaviour. Nursing Standard, 32(26), 51–63. 10.7748/ns.2018.e10969 29465213

[jocn17218-bib-0013] Hanson, G. C. , Perrin, N. A. , Moss, H. , Laharnar, N. , & Glass, N. (2015). Workplace violence against homecare workers and its relationship with workers health outcomes: A cross‐sectional study. BMC Public Health, 15(1), 11. 10.1186/s12889-014-1340-7 25595487 PMC4308913

[jocn17218-bib-0014] Ipsos . (2022). Ipsos Global Health Service Monitor 2022. https://www.ipsos.com/en‐uk/3‐5‐globally‐say‐their‐healthcare‐system‐overstretched

[jocn17218-bib-0015] Kim, S. , Gu, M. , & Sok, S. (2022). Relationships between violence experience, resilience, and the nursing performance of emergency room nurses in South Korea. International Journal of Environmental Research and Public Health, 19(5), 2617. 10.3390/ijerph19052617 35270308 PMC8910310

[jocn17218-bib-0016] Lincoln, Y. S. , & Guba, E. G. (1985). Naturalistic inquiry. SAGE.

[jocn17218-bib-0017] Liu, J. , Gan, Y. , Jiang, H. , Li, L. , Dwyer, R. , Lu, K. , Yan, S. , Sampson, O. , Xu, H. , Wang, C. , Zhu, Y. , Chang, Y. , Yang, Y. , Yang, T. , Chen, Y. , Song, F. , & Lu, Z. (2019). Prevalence of workplace violence against healthcare workers: A systematic review and meta‐analysis. Occupational and Environmental Medicine, 76(12), 927–937. 10.1136/oemed-2019-105849 31611310

[jocn17218-bib-0018] Luthar, S. S. , Cicchetti, D. , & Becker, B. (2000). The construct of resilience: A critical evaluation and guidelines for future work. Child Development, 71(3), 543–562. 10.1111/1467-8624.00164 10953923 PMC1885202

[jocn17218-bib-0019] Malterud, K. , Siersma, V. D. , & Guassora, A. D. (2016). Sample size in qualitative interview studies: Guided by information power. Qualitative Health Research, 26(13), 1753–1760. 10.1177/1049732315617444 26613970

[jocn17218-bib-0020] Morris, J. , Schlepper, L. , Dayan, M. , Jefferies, D. , Maguire, D. , Merry, L. , & Wellings, D. (2023). Public satisfaction with the NHS and social care in 2022. https://www.kingsfund.org.uk/publications/public‐satisfaction‐nhs‐and‐social‐care‐2022

[jocn17218-bib-0021] NHS England . (2021). Violence prevention and reduction standard. https://www.england.nhs.uk/wp‐content/uploads/2020/12/B0319‐Violence‐Prevention‐Reduction‐Standards.pdf

[jocn17218-bib-0022] NHS England . (n.d.). Violence prevention and reduction. Retrieved September 11, 2023, from https://www.england.nhs.uk/supporting‐our‐nhs‐people/health‐and‐wellbeing‐programmes/violence‐prevention‐and‐safety/

[jocn17218-bib-0023] Occupational Safety and Health Administration (OSHA) . (2015). Workplace violence in healthcare: Understanding the challenge. https://www.osha.gov/sites/default/files/OSHA3826.pdf

[jocn17218-bib-0024] Oxtoby, K. (2021). Another epidemic: Abuse and violence towards doctors from patients and the public. BMJ, 372, n739. 10.1136/bmj.n739 33762225

[jocn17218-bib-0025] Pina, D. , Llor‐Zaragoza, P. , López‐López, R. , Ruiz‐Hernández, J. A. , Puente‐López, E. , Galián‐Munoz, I. , & Martínez‐Jarreta, B. (2022). Assessment of non‐physical user violence and burnout in primary health care professionals. The modulating role of job satisfaction. Frontiers in Public Health, 10, 777412. 10.3389/fpubh.2022.777412 35186835 PMC8854207

[jocn17218-bib-0026] Pines, R. , Giles, H. , & Watson, B. (2021). Managing patient aggression in healthcare: Initial testing of a communication accommodation theory intervention. Psychology of Language and Communication, 25(1), 62–81.

[jocn17218-bib-0027] Pines, R. , Myers, K. K. , & Giles, H. (2023). Healthcare Professionals' emotional labor and management of workplace violence with underserved patients in the safety net context. Health Communication, 1–10. 10.1080/10410236.2023.2226307 37331974

[jocn17218-bib-0028] Pompeii, L. A. , Schoenfisch, A. , Lipscomb, H. J. , Dement, J. M. , Smith, C. D. , & Conway, S. H. (2016). Hospital workers bypass traditional occupational injury reporting systems when reporting patient and visitor perpetrated (type II) violence: Workers bypass traditional reporting systems. American Journal of Industrial Medicine, 59(10), 853–865. 10.1002/ajim.22629 27409575

[jocn17218-bib-0029] Richardson, S. K. , Grainger, P. C. , Ardagh, M. W. , & Morrison, R. (2018). Violence and aggression in the emergency department is under‐reported and under‐appreciated. The New Zealand Medical Journal, 131(1476), 50–58. 29879726

[jocn17218-bib-0030] Ritchie, J. , & Spencer, L. (1994). Qualitative data analysis for applied policy research. In B. Bryman & R. Burgess (Eds.), Analyzing qualitative data (pp. 173–194). Routledge.

[jocn17218-bib-0031] Rossi, M. F. , Beccia, F. , Cittadini, F. , Amantea, C. , Aulino, G. , Santoro, P. E. , Borrelli, I. , Oliva, A. , Ricciardi, W. , Moscato, U. , & Gualano, M. R. (2023). Workplace violence against healthcare workers: An umbrella review of systematic reviews and meta‐analyses. Public Health, 221, 50–59. 10.1016/j.puhe.2023.05.021 37406450

[jocn17218-bib-0032] Sammut, D. , Hallett, N. , Lees‐Deutsch, L. , & Dickens, G. L. (2022). A systematic review of violence risk assessment tools currently used in emergency care settings. Journal of Emergency Nursing, 49(3), 371–386. e5. 10.1016/j.jen.2022.11.006 36585335

[jocn17218-bib-0033] Somani, R. , Muntaner, C. , Hillan, E. , Velonis, A. J. , & Smith, P. (2021). A systematic review: Effectiveness of interventions to de‐escalate workplace violence against nurses in healthcare settings. Safety and Health at Work, 12(3), 289–295. 10.1016/j.shaw.2021.04.004 34527388 PMC8430427

[jocn17218-bib-0034] Tong, A. , Sainsbury, P. , & Craig, J. (2007). Consolidated criteria for reporting qualitative research (COREQ): A 32‐item checklist for interviews and focus groups. International Journal for Quality in Health Care, 19(6), 349–357. 10.1093/intqhc/mzm042 17872937

[jocn17218-bib-0035] Van Heugten, K. (2013). Resilience as an underexplored outcome of workplace bullying. Qualitative Health Research, 23(3), 291–301. 10.1177/1049732312468251 23211611

[jocn17218-bib-0036] Weiner, B. (1986). An attributional theory of motivation and emotion. Springer US. 10.1007/978-1-4612-4948-1 3903815

[jocn17218-bib-0037] Winkel, A. F. , Honart, A. W. , Robinson, A. , Jones, A.‐A. , & Squires, A. (2018). Thriving in scrubs: A qualitative study of resident resilience. Reproductive Health, 15(1), 53. 10.1186/s12978-018-0489-4 29587793 PMC5869777

[jocn17218-bib-0038] Wolf, L. A. , Delao, A. M. , & Perhats, C. (2014). Nothing changes, nobody cares: Understanding the experience of emergency nurses physically or verbally assaulted while providing care. Journal of Emergency Nursing, 40(4), 305–310. 10.1016/j.jen.2013.11.006 24439244

[jocn17218-bib-0039] World Health Organization, & International Labour Organization . (2021). COVID‐19: Occupational health and safety for health workers: Interim guidance, 2 February 2021 (WHO/2019‐nCoV/HCW_advice/2021.1). https://www.who.int/publications‐detail‐redirect/WHO‐2019‐nCoV‐HCW_advice‐2021‐1

[jocn17218-bib-0040] Yao, X. , Shao, J. , Wang, L. , Zhang, J. , Zhang, C. , & Lin, Y. (2021). Does workplace violence, empathy, and communication influence occupational stress among mental health nurses? International Journal of Mental Health Nursing, 30(1), 177–188. 10.1111/inm.12770 32808483

[jocn17218-bib-0041] Zhan, Y. , Kim, S. K. , Zhou, L. , Xie, B. , Li, Y. , Wen, B. , & Nie, L. (2019). Patient violence and health professionals' occupational outcomes in China: A time‐lagged survey study. International Journal of Nursing Studies, 94, 120–130. 10.1016/j.ijnurstu.2018.11.010 30951987

[jocn17218-bib-0042] Zhang, J. , Zheng, J. , Cai, Y. , Zheng, K. , & Liu, X. (2021). Nurses' experiences and support needs following workplace violence: A qualitative systematic review. Journal of Clinical Nursing, 30(1–2), 28–43. 10.1111/jocn.15492 32936970

[jocn17218-bib-0043] Zhong, X. F. , & Shorey, S. (2022). Experiences of workplace violence among healthcare workers in home care settings: A qualitative systematic review. International Nursing Review, 70(4), 596–605. 10.1111/inr.12822 36580395

