# Supplementary material for: Exploring staff experiences and perceptions of patient‐perpetrated violence in hospital settings: A qualitative study
Source: J Clin Nurs. 2024 May 19;35(5):2483–95. doi: 10.1111/jocn.17218 (PMC13068164; doi:10.1111/jocn.17218)
Supplement: Supplementary file 2 — Data S2. [file JOCN-35-2483-s003.docx]

**Supplementary File 2: Interview guide for patient-facing staff**

***Participant characteristics***

1. How long have you been working in your role?
2. How long have you been working in [your clinical area]?

***Experience/incident***

1. Would you begin by describing an incident of violence that you experienced or witnessed?

*Prompts:*

- *What was the situation in the clinical area (and/or the area in which violence occurred) at the time [contextual]?*
- *How did you react or what were your immediate actions to the situation [response]?*
- *Did you [in any way] sense that there was a violent or aggressive situation evolving?*
- *At the time of the incident, were any colleagues around you aware of a situation developing?*
- *How long had the patient been in hospital [where along the care pathway, e.g. admission, discharge]?*

1. Were you aware of any previous episodes of violence linked to this patient?
2. In general [not limited to the experience already discussed], do you think there is any way of knowing/communicating in advance that a violent incident might occur [e.g. through handover, clinical presentation, risk factors, observable behaviours]?
3. When patients are transferred from other clinical areas, in your experience, do you find that risk of violence is communicated? [explicitly or implicitly, including violence characteristics]
   - *If no, what difference might it make if you were prepared for potential violence/aggression?*
   - *If yes, do you believe doing this is beneficial? [e.g. preparing staff, planning ahead, staff behaving differently, reduced incidence of actual violence/aggression]*
4. Has the way you deal with [violent/escalating] patients or situations changed since this incident and/or other incidents?

***Closing stage of interview***

1. What are your thoughts on using a violence risk tool for screening all patients in [your clinical area]?

- *What features would be important for a violence risk assessment tool in [your clinical area]? [e.g. brief, paper-based or computer-based, automatic prompts, to be completed once or repeatedly per patient, management matrix]*
- *What could be done to maximise staff acceptance and use of a risk assessment tool in [your clinical area]?*

1. Is there anything you would like to ask?

*Share telephone numbers of the research team for further contact.*
