# Supplementary material for: Exploring staff experiences and perceptions of patient‐perpetrated violence in hospital settings: A qualitative study
Source: J Clin Nurs. 2024 May 19;35(5):2483–95. doi: 10.1111/jocn.17218 (PMC13068164; doi:10.1111/jocn.17218)
Supplement: Supplementary file 3 — Data S3. [file JOCN-35-2483-s001.docx]

**Supplementary File 3: Interview guide for service leads**

***Participant characteristics***

1. Please describe your role.
2. How long have you been working in this role?
3. Have you experienced any encounters of violence directed towards you?

***Opinions/attitudes/perceptions***

1. What do you think are the main causes violence towards staff? [e.g. medications, pre-existing conditions]
2. What types of behaviour could be early indications that someone might become violent or verbally aggressive?
3. In your opinion, can staff behaviours or attitudes precipitate patient aggression/violence in any way?
4. When new services are planned or existing services expanded, is the prevention of violence or aggression taken into consideration?
5. Do you think there is a particular problem with violent and aggressive patients in your service?
6. What are the types of strategies you use to diffuse [de-escalate] violence and aggression [not referring to calling security]?

***Closing stage of interview***

1. What are your thoughts on using a violence risk tool for screening all patients in [your clinical area]?

*Prompts:*

- *Do you think staff would be accepting of a violence risk assessment tool for use in [your clinical area]? Why or why not?*
- *What could be done to maximise staff acceptance and use of a risk assessment tool in [your clinical area]?*

1. Is there anything you would like to ask?

*Share telephone numbers of the research team for further contact.*
