# Supplementary material for: Exploring staff experiences and perceptions of patient‐perpetrated violence in hospital settings: A qualitative study
Source: J Clin Nurs. 2024 May 19;35(5):2483–95. doi: 10.1111/jocn.17218 (PMC13068164; doi:10.1111/jocn.17218)
Supplement: Supplementary file 4 — Data S4. [file JOCN-35-2483-s004.docx]

**Supplementary File 4: Framework development**

| **Initial themes** | **Initial categories** | **Core concepts** | **Final themes** |
| --- | --- | --- | --- |
| Precursors/risk factors for violence | Patient-related risk factors | Attributions for patient behaviour (Attribution Theory) The different 'types' of aggressive patient Violence predictability Recognition of triggers and early warning signs | Violence as (un)predictable |
|  | Staff-related risk factors |  |  |
|  | Environmental risk factors |  |  |
|  | Proactive action/de-escalation strategies |  |  |
| Attitudes and culture | Societal attitudes and/or cultural factors | The distribution of power and accountability Acceptance culture The 'distance' between senior management and frontline staff Feeling powerless (small cogs in a big machine) Feeling (un)seen and (un)supported The structural problems precipitating violence | Violence as (un)preventable |
|  | NHS/organisation/department attitudes |  |  |
|  | Staff attitudes |  |  |
| Responses to and/or consequences of violence | Staff emotional responses | The relationship between experience and tolerance Different degrees of expertise Staff 'resilience' A vicious cycle Attributions for staff behaviours and/or responses (Attribution Theory) | The cumulative toll of violence |
|  | Staff actions in response to violence |  |  |
|  | Managerial/organisational responses |  |  |
|  | Consequences |  |  |
